# Avulsion of the Penis

**Published:** 1907-06

**Authors:** 


### AVULSION OF THE PENIS.

Biondi (La Clinica Moderna) reports the case of a man, aged 64, whose penis was pulled off by an energetic young woman with whom he was attempting to have sexual intercourse against her will. The case came into the courts, and as it was of considerable forensic interest as to the possibility of such a thing occurring, experiments were conducted on the cadaver. It was found the force necessary to avulse the penis, in a flaccid state was greater than that possessed by an ordinary person. But in the erect state, which was accomplished on the cadaver by the injection of sodium chloride solution direct into the corpus cavernosum, the resistance, which was chiefly due to the tunica albuginea, was very much reduced, and quite within the average strength of a woman.

